# Evaluating the Role of Mechanical Bowel Preparation in Anterior Resection Through a Prospective Randomized Single-Blinded Trial

**DOI:** 10.7759/cureus.59784

**Published:** 2024-05-07

**Authors:** Akshay Bavikatte, Sudheer OV, Unnikrishnan G

**Affiliations:** 1 Colorectal Surgery, West Suffolk Hospital, Bury St Edmunds, GBR; 2 General and Colorectal Surgery, Amrita Institute of Medical Sciences, Kochi, IND

**Keywords:** post-operative morbidity, anastomotic leak, surgical wound infection, colorectal cancer, mechanical bowel prepration

## Abstract

Introduction

Mechanical bowel preparation (MBP) before colorectal surgery is a common practice to reduce bacterial levels and infection. However, recent studies and data analyses have shown that this practice may increase the incidence of postoperative septic complications. Limited information is available regarding MBP for rectal surgeries. Our study aimed to examine the impact of MBP on postoperative outcomes in patients undergoing anterior resection with primary anastomosis for rectal cancer in a single-blinded, single-center, prospective, randomized trial.

Materials and methods

Data were collected between September 2013 and December 2015 at the Amrita Institute of Medical Sciences, Kochi, India. All patients scheduled for elective anterior resection with primary anastomosis for cancer between 5 cm and 15 cm were included in the study. All patients were randomized into the MBP and non-MBP groups after obtaining consent using a computer-based randomizer. The MBP group underwent bowel preparation with polyethylene glycol 24 hours before the operation and received sodium phosphate rectal enemas the night before the procedure. In the non-MBP group, only dietary restriction with a low-residue diet for 48 hours was recommended. Laparoscopic and open surgeries were performed. A contrast enema with barium was performed on all patients on postoperative days 6-8 to detect an anastomotic leak. Our primary endpoint was to assess the rate of anastomotic leakage between the two groups. The secondary endpoints were surgical site infection and postoperative morbidity.

Results

A total of 78 patients were recruited in the trial, and 18 were excluded because the surgery was the Hartmann procedure or abdominal perineal resection. The remaining 60 patients were divided equally into the MBP and non-MBP groups. No clinically significant disparities were evident between the groups concerning the preoperative prognosticators of anastomotic leak. Among the cohort, anastomotic leakage occurred in eight patients, representing a 13.3% incidence. Remarkably, within this subset, seven patients (23.3%) were attributed to the non-MBP cohort, whereas only one patient (3.3%) belonged to the MBP group. These findings demonstrated a statistically noteworthy discrepancy. The two groups had no statistically significant difference in surgical site infection and postoperative morbidity.

Conclusion

Our study suggests the benefit of preoperative MBP in sphincter-preserving rectal surgery to reduce the anastomotic leak rate. Additionally, incorporating large-scale studies and meta-analyses could enhance the robustness of our conclusions.

## Introduction

Preoperative mechanical bowel preparation (MBP) has been the standard practice in colorectal cancer surgery since the 20th century, but its routine use began in 1960 [1]. The practice is believed to reduce the risk of postoperative infectious complications and anastomotic leakage; however, no evidence supports this [2]. MBP has advantages such as reducing the bacterial content in the colon, making it easier to manipulate the colon during laparoscopic surgery, and aiding in the palpation of lesions. However, it also has potential disadvantages such as patient discomfort, electrolyte imbalance, increased hospital stays, and the need for additional agents to improve taste. Recent meta-analyses and systematic reviews have concluded that MBP could be omitted before elective colorectal surgery because it does not affect the complication rate [3,4]. Some studies have found that MBP increases the risk of anastomotic leakage due to intraoperative septic complications exacerbated by liquid stools [5]. However, most studies have excluded patients with rectal cancer from their data collection because rectal cancer surgeries have unique factors such as the level of anastomosis, the need for diverting stomas, and preoperative chemoradiotherapy [6].

Rationale for the study

In 2010, Bretagnol et al. along with the French Research Group of Rectal Cancer Surgery (GRECCAR) conducted the first randomized, multi-center, single-blinded study with 178 patients to investigate postoperative outcomes following sphincter-saving rectal surgery [7]. The study's findings established that rectal surgery performed without MBP posed a higher risk of overall and infectious morbidities. However, more data about this subject must be collected in the existing literature. Consequently, our objective was to evaluate the feasibility of undertaking rectal cancer surgery without preoperative MBP.

## Materials and methods

This was a prospective, single-blinded, randomized controlled trial was conducted from December 2013 to December 2016 in the department of surgical gastroenterology at Amrita Institute of Medical Sciences in Kochi, India. The ethics committee (ECR/129/Inst/KL/2013/rr-16) approval was obtained before the study was implemented. The trial was registered with the Clinical Trial Registry of India (CTRI/2014/08/01950).

Inclusion and exclusion criteria

All consenting patients aged ≥ 18 years with biopsy-proven rectal cancer who underwent elective open or laparoscopic sphincter-preserving surgery during the study period were included. Patients presenting with tumors situated more than 15 cm above or within 5 cm below the anal verge upon rigid sigmoidoscopy, those necessitating abdominoperineal resection due to rectal tumors, and those with clinically significant obstructive rectal cancer requiring emergency surgery were excluded from the study. Additionally, patients diagnosed with stage IV rectal cancer based on the American Joint Committee on Cancer (AJCC) Tumor-Node-Metastasis (TNM) Staging System, 6th edition [8] were also excluded. Patients presenting with synchronous adenocarcinomas and/or gastrointestinal conditions such as inflammatory bowel disease or familial polyposis necessitating extensive colonic surgery were excluded. Patients with a medical history indicating clinically significant renal disease (serum creatinine levels exceeding 1.5 mg/dl) or cardiac conditions (ejection fraction below 45%) were also excluded. Additionally, the study cohort did not include individuals who remained inadequately prepared despite receiving polyethylene glycol solution but still passing liquid stools rather than clear bowel movements before surgery.

Sample size estimation

Following prior research findings examining the incidence rates of anastomotic leakage and wound complications within two distinct treatment cohorts, namely those receiving MBP and those without such intervention, it was determined that achieving a sample size of at least 240 patients per group was imperative. This sample size was deemed essential to ensure an 80% statistical power with a 95% confidence interval (CI) for robustly assessing the occurrence of anastomotic leakage. Similarly, a sample size of 400 patients per group was deemed necessary for evaluating wound complications. However, logistical constraints limited the allocation to a maximum of 60 cases, with only 30 patients per group, and the absence of relevant literature from the Indian context posed challenges. Consequently, the study was structured as a pilot investigation to address these limitations and provide insights for future research.

Study protocol

The study protocol included obtaining clearance from the Institutional Ethical Committee before commencement and obtaining informed consent from all patients before their inclusion in the trial. Patients in both groups were evaluated using the standard protocol followed in our institution, which included clinical examination, rigid sigmoidoscopy, colonoscopy with biopsy, endorectal ultrasound, multidetector computed tomography (MDCT) abdomen, magnetic resonance imaging (MRI) abdomen, and chest X-ray to assess the stage of the disease. The patients were discussed in a multidisciplinary tumor board, and the decision for multimodal therapy was made based on the AJCC TNM 6th edition classification.

According to the established protocol, all patients were instructed on a low-residue diet 48 hours before the procedure and admitted one day before surgery. The MBP group was given two standard packs (137.15 g) of polyethylene glycol solution to be dissolved in 4 L of boiled and cooled water, and rectal sodium phosphate enema was given the night before surgery. The polyethylene glycol solution was consumed within eight hours, starting at 9 am the day before surgery. The diet of patients in the MBP group will be restricted to two of 10 clear liquids only following the initiation of the bowel preparation solution. The non-MBP group patients were asked to continue their low-residue diets.

At 10 pm on the night before surgery, both groups of patients were required to refrain from eating and drinking. The operating surgeon was unaware of the patient's arm until the morning of the operation. All patients received preoperative intravenous antibiotics consisting of 1.5 g of cefuroxime and 500 mg of metronidazole, administered during anesthetic induction, repeated after eight hours, and continued for 24 hours after surgery routinely. Further antibiotics were administered if there was any indication of sepsis or contamination during the surgery. The postoperative course of all patients was closely monitored for return of bowel function, symptoms of abdominal or non-abdominal infections, and wound infection. Diatrizoate meglumine (Gastrografin) contrast enema fluoroscopy was performed on all patients in the sixth to eighth postoperative periods to detect the anastomotic leak.

Surgical technique

In patients undergoing preoperative chemoradiotherapy, surgery was performed six to eight weeks after completing irradiation. All patients underwent either midline laparotomy or laparoscopic surgery, with the technique used at their discretion. Intraoperative rectal washout distal to the tumor was performed using a 28-French gauge catheter irrigated with 500 ml of sterile water. Both methods involved high ligation of the inferior mesenteric artery and total mesorectal excision of the rectal tumor. Rectal dissection was performed 5 cm below the lower edge of the tumor for the upper third of the rectum and to the pelvic floor for mid- and low rectal tumors, with total mesorectal excision and nerve preservation. Reconstruction was performed via colorectal anastomosis using a circular stapler size 29 (CDH; Ethicon Endo-Surgery, Cincinnati, Ohio, United States). Doughnuts were always inspected for completeness, and transanal fluid instillation tests were performed to assess the anastomotic integrity during surgery. Pelvic drains were placed behind the anastomosis.

All patients received a peritoneal wash with 5 l of normal saline, and subcutaneous tissue was washed with a pulsatile lavage system. The decision for an ileostomy was at the discretion of the surgeon. If performed, it was reversed within six to eight weeks unless there was no evidence of anastomotic leakage on the rectal contrast enema or MDCT scan of the abdomen.

Primary endpoint

Anastomotic Leakage

The International Study Group of Rectal Cancer (ISREC) defines anastomotic leakage following anterior resection of the rectum entails communication between the intra- and extraluminal compartments due to an integrity defect in the intestinal wall at the anastomosis or anorectal reservoir (such as a J-pouch or transverse coloplast) [9]. Additionally, the presence of a pelvic abscess in close proximity to an anastomosis is recommended to be categorized as leakage, irrespective of whether its point of origin is identifiable. Grade A anastomotic leakage requires no active therapeutic intervention, Grade B anastomotic leakage necessitates active therapeutic intervention but can be managed without re-laparotomy, and Grade C anastomotic leakage necessitates relaparotomy. All patients in both groups underwent rectal contrast enema examination on the fifth or sixth postoperative day to assess anastomotic integrity.

Secondary endpoint

Surgical Site Infection (SSI)

SSI refers to a wound that requires complete or partial opening to drain purulent material or an erythematous area that involves initiating antibiotics and postoperative morbidity according to the Clavien-Dindo classification [10].

Statistical analysis

Statistical significance testing assessed differences in percentages for categorical study variables using Chi-square tests with correction factors. For measurable variables between the two groups, the student's t-test or Mann-Whitney U-test was employed based on the normality of the data distribution. A significance level of p < 0.05 was utilized to determine statistical significance. Data analysis was performed using IBM SPSS Statistics for Windows, Version 20.0 (Released 2011; IBM Corp., Armonk, New York, United States).

## Results

**Figure 1:**
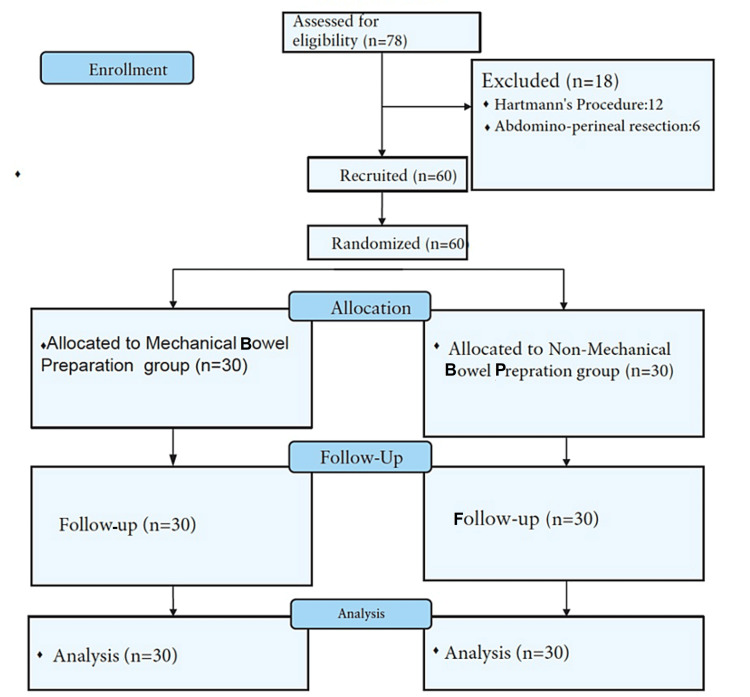
CONSORT flow diagram of study recruitment CONSORT: Consolidated Standards of Reporting Trials

The Consolidated Standards of Reporting Trials (CONSORT) flow diagram represents study recruitment (Figure 1) [11]. A total of 78 patients were initially recruited for the study, of which 12 were excluded because of intraoperative complications such as improper stapling, insufficient length of the colon, or anastomotic leak identified intra-operatively following stapling that led to Hartmann's procedure, and six were excluded because their procedure turned out to be an abdominoperineal resection (APR) due to the position of the tumor intra-operatively that was lower than the preoperative investigation. The remaining 60 patients of which the majority were males (n=37, 61.6%) were included in this analysis.

The median age of the patients was 62.5 years (range of 40-80 years), and the mean BMI was 27.3 kg/m^2^, with a range of 18-40 kg/m^2^. The tumor location was divided into high rectal tumors (10-15 cm from the anal verge) and low rectal tumors (5-10 cm from the anal verge), and the distribution of tumor locations was comparable between the two groups. In this study, 33 (55%) of 60 patients received long course. The demographics of the patients are given according to the MBP and non-MBP groups in Table 1.

**Table 1:** Study population demographics (N=60) P values were calculated using Student t-test MBP: mechanical bowel preparation; BMI: body mass index; ASA: American Society of Anesthesiologists; AJCC: American Joint Cancer Committee on Cancer

Study Population (N)	MBP Group (N=30)	Non-MBP Group (N-30)	p-value
Age (years), median (range)	62.5 (40-80)	63 (40-80)	>1
Male Sex, n (%)	18 (60%)	19 (63%)	>1
Body mass index (kg/m^2^), median (range)	26 (18-40)	26 (18-40)	0.225
Distribution of Tumor location			
High (11-15 cm from the anal verge), n (%)	13 (43.3%)	17 (56%)	>1
Low (5-10 cm from the anal verge), n (%)	16 (54%)	14 (50%)	>1
Patients receiving neoadjuvant chemoradiotherapy, n (%)	18 (60%)	15 (50%)	0.6
ASA Grade 2, %	68%	58%	0.7
AJCC staging of carcinoma of the rectum, n (%)			
Stage 1	18 (53%)	12 (36%)	0.6
Stage 2	4 (12%)	7 (21%)	
Stage 3	8 (26%)	11 (33%)	

Intraoperative and postoperative details

The operative times in the MBP and non-MBP groups, with mean durations of 283.40 and 297.83 minutes, respectively, showed no statistically significant difference. Similarly, 38 patients (63%) underwent laparoscopic anterior resection, with no significant difference between the two groups. There was also no statistically significant difference in the rate of laparoscopic conversion between the two groups. Moreover, there was no statistically significant difference in the postoperative ileus and length of hospital stay between the two groups, as shown in Table 2.

**Table 2:** Perioperative data MBP: mechanical bowel preparation P values were calculated using a chi-square test for categorical data and Student t-test for others

	MBP	Non-MBP	P-value
Operative time (minutes), mean	283.4	297.83	0.53
Laparoscopic surgery, n (%)	18 (60%)	20 (66%)	0.54
Diverting ileostomy, n (%)	15 (50%)	8 (26%)	0.56
Laparoscopic conversion, n (%)	4 (30%)	2 (20%)	0.58
Duration of postoperative ileus (days), mean	3.13	3.16	0.53
Length of hospital stay (days), mean	8.6	9	0.57

Anastomotic leak

The severity of the anastomotic leak was assessed using the grading system recommended by ISREC. Our study identified anastomotic leak clinically by observing patients with evidence of a leak on cross-sectional examination or via fluoroscopy conducted using a Gastrografin enema as a part of the study protocol. The overall anastomotic leak rate was 13.3% (n=8). The number of patients with anastomotic leaks was higher in the non-MBP group (n=7, 23.3%) compared to the MBP group (n=1, 3.3%) and this difference was statistically significant (Table 3). In the MBP group, only one patient had an anastomotic leak, which was clinically detected, while seven patients had an anastomotic leak in the non-MBP group, three of which were detected clinically and the remaining four were detected via fluoroscopy (Table 4). Further subgroup analysis revealed that the majority of patients had Grade C leaks that required laparotomy.

**Table 3:** Anastomotic leak between the two groups MBP: mechanical bowel preparation P values were calculated using the Chi-square test

Group	Anastomotic Leak		P -Value
	Yes, n (%)	No, n (%)	
MBP (N=30)	1 (3.3%)	29 (96.7%)	0.0023
Non-MBP (N=30)	7 (23.3%)	23 (76.7%)	

**Table 4:** Detection of anastomotic leak (severity of the anastomotic leak) MBP: mechanical bowel preparation

Group	Leaks Detected Clinically, n (%)	Leaks Detected Radiologically, n (%)	Total
Non-MBP	3 (42%)	4 (58%)	7
MBP	1 (100%)	0 (0%)	1

SSI

Our secondary objective was to evaluate the risk of SSI in both groups within 30 postoperative days. The overall incidence of SSI was 26% (n=16). The difference in the number of SSI in the non-MBP (n=10, 33%) and MBP (n=6, 20%) groups was not statistically significant (Table 5). Of the patients who had SSI, the majority (n=14, 87%) experienced superficial incisional SSIs, while two patients (13%) from each group required laparotomy due to a burst abdomen.

**Table 5:** Surgical site infection between the two groups MBP: Mechanical bowel preparation; n: number of patients; P values were calculated using Chi-square test.

	Patient Group	Surgical Site Infection		
		Yes (n, %)	No (n, %)	P- Value
Surgical Site Infection	MBP patients	6 (20%)	24 (80%)	0.243
	Non-MBP patients	10 (33.3%)	20 (66%)	

Postoperative morbidity

Our study observed a comprehensive postoperative morbidity rate of 45%, affecting 27 patients, 15 of whom were in the non-MBP group (50%) and 12 were in the MBP group (40%). The distribution of postoperative morbidity among the two groups is presented in Table 6. The non-MBP group had a higher incidence of grade 3 morbidity; however, the difference was insignificant.

**Table 6:** Postoperative morbidity in the two groups according to Clavien-Dindo classification MBP: mechanical bowel preparation

Groups	Postoperative morbidity	Grade 1	Grade 2	Grade 3	Grade 4	Grade 5
MBP (N=30)	Number of Patients (N=12)	4	1	4	2	1
	Percentage (40%)	13.3%	3.35%	13.3%	6.7%	3.35%
Non-MBP (N=30)	Number of Patients (N=15)	0	6	5	4	0
	Percentage (50%)	0%	20%	16.7%	13.3%	0%
Total	Number of Patients (N=27)	3	7	9	6	1
	Percentage (45%)	5.3%	11.8%	15.5%	10.7%	1.7%

## Discussion

Rectal cancer is a significant health concern worldwide. In India, the incidence of rectal cancer has shown mixed trends, with some studies indicating stable or decreasing trends in specific regions [12]. Despite having a lower overall incidence rate of colorectal cancer compared to those in the Western world, there has been a notable increase in the incidence of rectal cancer in both men and women in India [13]. Moreover, there has been a substantial rise in the occurrence of colorectal cancer among younger adults aged 20-49 years, representing a relative increase of 30% over the span of a decade [14].

MBP has long been an exciting topic in colorectal surgery, especially rectal cancer surgery. The Enhanced Recovery After Surgery (ERAS) Society recommends that MBP may be beneficial for rectal surgery but is not routinely recommended for colonic surgery [15]. Our study aimed to evaluate the necessity of preoperative MBP for rectal cancer surgery owing to the lack of consensus. To the best of our knowledge, the current randomized controlled trial is the first in India involving only patients undergoing rectal cancer surgery.

In a study by the Korean laparoscopic colorectal surgery study group, 1609 patients from 11 institutions were analyzed for the risk factors of anastomotic leak after laparoscopic rectal cancer surgery [16]. Male sex, low tumor level, preoperative chemoradiotherapy, and absence of diverting stomas were identified as independent predictors of anastomotic leak. In our study, there was no clinically significant difference between the two groups regarding age, sex, body mass index, tumor location, preoperative chemoradiation, and creation of diverting stomas.

In a study by Bretagnol et al. in 2010, the French Research Group of Rectal Cancer Surgery (GRECCAR) trial was the first randomized multicenter trial of MBP for rectal cancer [7]. A total of 178 patients were included in their study, with 89 patients in each group. The overall rate of anastomotic leak was 14%, with no significant difference between the MBP and non-MBP groups (19% vs. 10%, p = 0.09). Although there was an increased incidence of anastomotic leak in the non-MBP group, the difference was not statistically significant. In 2015, Morris et al. analyzed 4999 patients from the American College of Surgeons-National Surgical Quality Improvement Program (ACS-NSQIP) database. They divided them into four groups based on their treatment: MBP with oral antibiotic preparation, oral antibiotic preparation only, MBP only, and no MBP. The overall rate of anastomotic leak was 4.1%, with no significant difference between the MBP and non-MBP groups (4.2% vs. 5.7%). MBP with oral antibiotic preparations had the lowest incidence of SSI, which was statistically significant [17]. In our study, the overall anastomotic leak rate was 13% (n=80), similar to that reported in the literature. However, the non-MBP group had a significantly higher rate of anastomotic leak than the MBP group (11.7% vs. 3%, p = 0.001).

The literature lacks a comprehensive investigation into the adequacy of bowel preparation preceding surgery and its potential correlation with anastomotic leak occurrence. Previous research on the efficacy of MBP in colorectal surgery has produced inconclusive results. Inadequate bowel preparation has been associated with similar leak rates between unprepared and poorly prepared patients, likely due to increased bowel content and compromised intraoperative handling [18]. In our study, all patients were admitted 48 hours before surgery, bowel preparation started 24 hours before surgery, and their effluent was evaluated. Except for two patients, all passed clear fluid rectally before surgery. These two poorly prepared patients were included in the analysis. Cultural factors, such as protein-rich and fiber-poor Western diets, may diminish the importance of bowel preparation compared to bulkier Indian diets [19]. The French GRECCAR trial suggested that preparation may influence pelvic sepsis severity, with non-MBP groups showing higher peritonitis rates post-leak, although this was not statistically significant [7]. In our study, the preparation status showed no significant impact on the severity of pelvic sepsis. This finding is possibly attributed to the low incidence of leaks and the predominance of well-prepared patients within the MBP group.

According to a systematic review conducted by Cong et al., the rate of asymptomatic leakage (Grade A) following anterior resection for rectal cancer was 2.57% [20]. The rate of leakage that required active intervention without re-laparotomy (Grade B) was 2.37%, whereas the rate of leakage that required re-laparotomy (Grade C) was 5.40%. In the current study, of the eight patients who experienced leakage, one (12.5%) was in Grade A. Three (37.5%) patients were in Grade B, and the leak was diagnosed on a routine Gastrografin enema performed on postoperative day 6. As there was no peritonitis, these patients were managed conservatively. Four (50%) patients were Grade C, and all leaks were diagnosed on postoperative day 2 or 3. All four patients exhibited signs of peritonitis that required laparotomy. Three patients underwent Hartmann's procedure, and one underwent a diverting ileostomy as there was relatively reduced stool content in the colon.

In a meta-analysis conducted by Wille-Jørgensen et al. in 2005, which included nine randomized controlled trials involving 791 patients receiving MBP and 803 receiving no preparation, the overall SSI rate was 8% (7.4% in MBP vs. 5.4% in non-MBP) [21]. This suggests that there may be a potential benefit of not using MBP in reducing SSI. However, a later analysis by Güenaga et al., who performed an updated Cochrane review of MBP in colorectal surgery that included 13 randomized controlled trials with 2390 patients allocated to MBP and 2387 to no preparation, found no significant difference in the rate of SSI between the two groups (9.6% in MBP vs. 8.3% in non-MBP) [22]. Similarly, the French GRECCAR trial, the only multicenter randomized study, also showed no difference in the incidence of SSI between the two groups (3% in MBP vs. 1% in non-MBP) [7].

In the current study, the SSI rate was 33.3% in the non-MBP group and 20% in the MBP group. One possible reason for the higher incidence of SSI could be the tropical humid climate, which may favor bacterial infections [23]. It is possible that the absence of preoperative oral antibiotics played a role in this outcome [24]. The higher incidence of SSI in the non-MBP group was not statistically significant. In Indian centers, this was not a standard practice [24], unlike recent studies that have shown positive results with preoperative oral antibiotics alone or in combination with MBP for preventing SSI [25].

To the best of our knowledge, to date, no studies have evaluated the impact of MBP in patients undergoing elective laparoscopic surgery. Despite this lack of evidence, some surgeons contend that MBP is necessary owing to the loss of tactile sensation, which can lead to difficulty locating small tumors accurately. Additionally, an unprepared bowel is considered heavier and more challenging to manipulate laparoscopically [26]. In our study, laparoscopic surgery was performed on 38 patients, constituting 63.3% of the cohort. Within the MBP group, 18 patients (60%) underwent laparoscopic procedures, while four individuals (30%) necessitated conversion from laparoscopic to open surgery, with the remainder undergoing open procedures. In contrast, among the non-MBP group, laparoscopic surgery was conducted in 20 patients (66%), and two cases (20%) necessitated the conversion to open surgery while the rest had open surgeries. There was no statistically significant difference in conversion rates between the MBP and non-MBP groups.

The French GRECCAR trial showed an increased overall postoperative major morbidity rate (Clavien-Dindo III or more), with 10% in the MBP group and 16% in the non-MBP group [7]. However, this difference was not clinically significant. Similarly, in our study, the overall incidence of postoperative morbidity was 27 (45%). The incidence of major morbidity was higher in the non-MBP group (50%) than in the MBP group (40%).

The strength of our study is that it is, according to the best of our knowledge, India's first randomized controlled MBP trial in rectal cancer surgery. Very few similar studies in the literature are noted worldwide. With the help of all consultant colorectal surgeons, we established a standardized protocol for perioperative and postoperative processes that would exclude operator bias. It was a single-blinded study where the consultant surgeon was unaware of the patients' arms until the day of the operation. The main limitation of the study is the sample size. To address this limitation, future studies should consider increasing the sample size to enhance the validity of our results. Alternatively, other methods such as meta-analysis or sensitivity analysis can combine the results of multiple studies to increase the statistical power.

## Conclusions

The current study suggests the benefit of preoperative MBP in sphincter-preserving rectal surgery to reduce the anastomotic leak rate. Additionally, incorporating larger-scale studies and meta-analyses could enhance the robustness of our conclusions.
